# Compressed Sensing Real-Time Cine Reduces CMR Arrhythmia-Related Artifacts

**DOI:** 10.3390/jcm10153274

**Published:** 2021-07-24

**Authors:** Benjamin Longère, Paul-Edouard Allard, Christos V Gkizas, Augustin Coisne, Justin Hennicaux, Arianna Simeone, Michaela Schmidt, Christoph Forman, Solenn Toupin, David Montaigne, François Pontana

**Affiliations:** 1 University of Lille, Inserm, CHU Lille, Institut Pasteur Lille, U1011—European Genomic Institute for Diabetes (EGID), F-59000 Lille, France; augustin.coisne@chru-lille.fr (A.C.); david.montaigne@chru-lille.fr (D.M.); francois.pontana@chru-lille.fr (F.P.); 2 CHU Lille, Department of Cardiovascular Radiology, F-59000 Lille, France; pauledouard.allard@chru-lille.fr (P.-E.A.); chgkizas@gmail.com (C.V.G.); justin.hennicaux@chru-lille.fr (J.H.); arianna.simeone@chru-lille.fr (A.S.); 3 MR Product Innovation and Definition, Magnetic Resonance, Siemens Healthcare GmbH, 91052 Erlangen, Germany; michaela.schmidt@siemens-healthineers.com (M.S.); christoph.forman@siemens-healthineers.com (C.F.); 4 Scientific Partnerships, Siemens Healthcare France, 93200 Saint-Denis, France; solenn.toupin@siemens-healthineers.com

**Keywords:** cardiac, heart, magnetic resonance, CMR, compressed sensing, real-time, fast imaging, arrhythmia, artifact

## Abstract

Background and objective: Cardiac magnetic resonance (CMR) is a key tool for cardiac work-up. However, arrhythmia can be responsible for arrhythmia-related artifacts (ARA) and increased scan time using segmented sequences. The aim of this study is to evaluate the effect of cardiac arrhythmia on image quality in a comparison of a compressed sensing real-time (CS_rt_) cine sequence with the reference prospectively gated segmented balanced steady-state free precession (Cine_ref_) technique regarding ARA. Methods: A total of 71 consecutive adult patients (41 males; mean age = 59.5 ± 20.1 years (95% CI: 54.7–64.2 years)) referred for CMR examination with concomitant irregular heart rate (defined by an RR interval coefficient of variation >10%) during scanning were prospectively enrolled. For each patient, two cine sequences were systematically acquired: first, the reference prospectively triggered multi-breath-hold Cine_ref_ sequence including a short-axis stack, one four-chamber slice, and a couple of two-chamber slices; second, an additional single breath-hold CS_rt_ sequence providing the same slices as the reference technique. Two radiologists independently assessed ARA and image quality (overall, acquisition, and edge sharpness) for both techniques. Results: The mean heart rate was 71.8 ± 19.0 (SD) beat per minute (bpm) (95% CI: 67.4–76.3 bpm) and its coefficient of variation was 25.0 ± 9.4 (SD) % (95% CI: 22.8–27.2%). Acquisition was significantly faster with CS_rt_ than with Cine_ref_ (Cine_ref_: 556.7 ± 145.4 (SD) s (95% CI: 496.7–616.7 s); CS_rt_: 23.9 ± 7.9 (SD) s (95% CI: 20.6–27.1 s); *p* < 0.0001). A total of 599 pairs of cine slices were evaluated (median: 8 (range: 6–14) slices per patient). The mean proportion of ARA-impaired slices per patient was 85.9 ± 22.7 (SD) % using Cine_ref_, but this was figure was zero using CS_rt_ (*p* < 0.0001). The European CMR registry artifact score was lower with CS_rt_ (median: 1 (range: 0–5)) than with Cine_ref_ (median: 3 (range: 0–3); *p* < 0.0001). Subjective image quality was higher in CS_rt_ than in Cine_ref_ (median: 3 (range: 1–3) versus 2 (range: 1–4), respectively; *p* < 0.0001). In line, edge sharpness was higher on CS_rt_ cine than on Cine_ref_ images (0.054 ± 0.016 pixel^−1^ (95% CI: 0.050–0.057 pixel^−1^) versus 0.042 ± 0.022 pixel^−1^ (95% CI: 0.037–0.047 pixel^−1^), respectively; *p* = 0.0001). Conclusion: Compressed sensing real-time cine drastically reduces arrhythmia-related artifacts and thus improves cine image quality in patients with arrhythmia.

## 1. Introduction

Cardiac magnetic resonance (CMR) is a major imaging modality for the assessment of left and right ventricular volumes and mass [1,2,3]. Moreover, it provides effective morphologic and kinetic assessment, including of the right ventricle which is not easily evaluated with ultrasounds due to its retrosternal location [4]. Multi-breath-hold segmented balanced steady state free precession (bSSFP) sequences are considered superior to gradient-echo imaging since they provide better endocardium delineation and reproducibility in a shorter scan time [5]. Retrospective electrocardiogram (ECG) gating requires the heart rate (HR) to be a regular periodic phenomenon as pieces of data are continuously acquired on multiple cardiac cycles, time-labelled and merged for the reconstruction of a whole cine slice, which is a weighted representation of successive heartbeats. It allows adapting the length of the acquisition window to the duration of the heartbeat during the continuous acquisition. This enables capturing of the complete cardiac cycle in segmented acquisitions. Typically, k-space interpolation or filtering is applied to retrospectively gate the acquired data to a reference heartbeat [6,7]. In the case of arrhythmia, artifacts occur since reconstruction is performed using incoming data from different frames of the cardiac cycle. Arrhythmia rejection algorithms can be applied with retrospective gating but may end in exceedingly long breath-holds. These arrhythmia-related artifacts (ARA) may be limited using prospectively triggered sequences by setting the acquisition window shorter than the briefest measured RR interval (time laps between two consecutive R peaks) [8]. However, this requires decreasing the number of k-space lines acquired per cardiac frame in order to preserve the widely accepted temporal resolution of 20 phases per cardiac cycle and misses to display the diastolic phases [9,10]. As a result of these adjustments, longer breath-holds and scan time are observed while the last phases of the cardiac cycle are not sampled.

Decreasing the amount of measured data is a simple way to reduce acquisition time. In recent years, compressed sensing was established as a powerful method to drastically reduce scan time [11,12,13,14]. This is achieved by highly undersampling k-space with a random sampling pattern. After Fourier transform, these acquired data result in noise-like, incoherent artifacts. These artifacts are compensated for in the final image with a non-linear iterative reconstruction exploiting the fact that medical images have a sparse representation. In combination with parallel imaging, acceleration rates can be achieved with CS that enable real-time cardiac cine imaging based on a balanced bSSFP readout with spatiotemporal resolution in a similar range to the reference (Cine_ref_) acquisitions [15].

Various CMR studies have evaluated real-time CS cine sequences in 1.5 and 3 Tesla magnetic resonance scanners showing promising results for the assessment of left and right ventricles, including in patients with atrial fibrillation [16,17,18,19,20,21]. However, image quality was not specifically assessed in patients with irregular HR. Based on the assumption that real-time CS cine (CS_rt_) could reduce ARA, our study aimed at evaluating its image quality as compared to the reference multi-breath-hold segmented bSSFP cine (Cine_ref_) in patients suffering from cardiac arrhythmia.

## 2. Materials and Methods

### 2.1. Study Population

From January 2019 to December 2019, 71 adult patients referred to our cardiovascular radiology department for CMR with concomitant arrhythmia during scanning were enrolled. Irregular HR was defined when the coefficient of variation of RR intervals (CV_RR_) was greater than 10% while scanning. The CV_RR_ was calculated as the ratio of the standard deviation to the mean of RR intervals’ durations which were obtained from digital imaging and communications in medicine (DICOM) fields. Patients under 18 years old, grown-up congenital heart disease, stress CMR, patients undergoing ECG retrogated CMR and patients with sinus rhythm were excluded. A graphic illustration of the study design is provided in Figure S1 (Supplementary Materials). The protocol was approved by our institutional ethics committee and patients gave informed consent. The study was approved by the French National Agency for the Safety of Medicines and Health Devices (ANSM; ID-RCB: 2017-A00852-51).

### 2.2. Imaging Protocol

CMR studies were performed on a 1.5 T scanner (MAGNETOM Aera, Siemens Healthcare, Erlangen, Germany). Every patient underwent two series of cine images: first, the reference prospectively triggered and segmented multi-breath-hold Cine_ref_ sequence; second, the prototype single-breath-hold real-time single-shot CS_rt_ cine sequence. Both acquisitions included one left ventricular (LV) and one right ventricular (RV) two-chamber slice, one four-chamber slice and a LV short-axis stack covering both ventricles with an 8 mm slice thickness and a 2 mm gap. Regarding the prospectively gated Cine_ref_ sequence, 20 phases of the cardiac cycle were acquired and the number of views per frame was set to reach this sampling rate. In single-shot CS_rt_ cine imaging, the data acquisition was performed in a single heartbeat. The acquisition was triggered by the R peak on the ECG. With adaptive triggering, the acquisition was stopped with the next R peak, which allowed capturing the complete cardiac cycle. However, for multi-slice acquisition it could lead to a variation in the number of cardiac phases acquired between different slices as the temporal resolution was fixed. Temporal interpolation was applied to generate an additional dataset with a fixed number of cardiac phases (*n* = 20). This dataset was used to quantify cardiac function using a dedicated post-processing software that required a fixed number of cardiac phases. To evaluate the CS_rt_ sequence in clinical conditions, 40 iterations were used to perform image recovery to maintain an acceptable reconstruction time. An additional phase contrast imaging (PCI) flow sequence was acquired on the aortic root. Segmented Cine_ref_ and CS_rt_ cine sequences parameters are available in Table 1.

### 2.3. Cine Images Quality Assessment

Image quality was evaluated in both groups using four indicators. First, the subjective overall image quality was evaluated using a subjective 4-point Likert scale (1: non diagnostic; 2: poor; 3: good; 4: excellent). Secondly, an objective image quality assessment was carried out based on standardized criteria adapted from the European CMR registry “LV-Function cine SSFP” section (referred to below as “EuroCMR score”) [22] (p. 3). Higher scores referred to more frequent artifact occurrence (Table 2).

Third, the proportion of short-axis slices affected by ARA in each stack of both sequences was calculated, referred to as ARA rate. ARA were defined as a blurring of all or a part of the LV wall borders [22].

**Figure 1:**
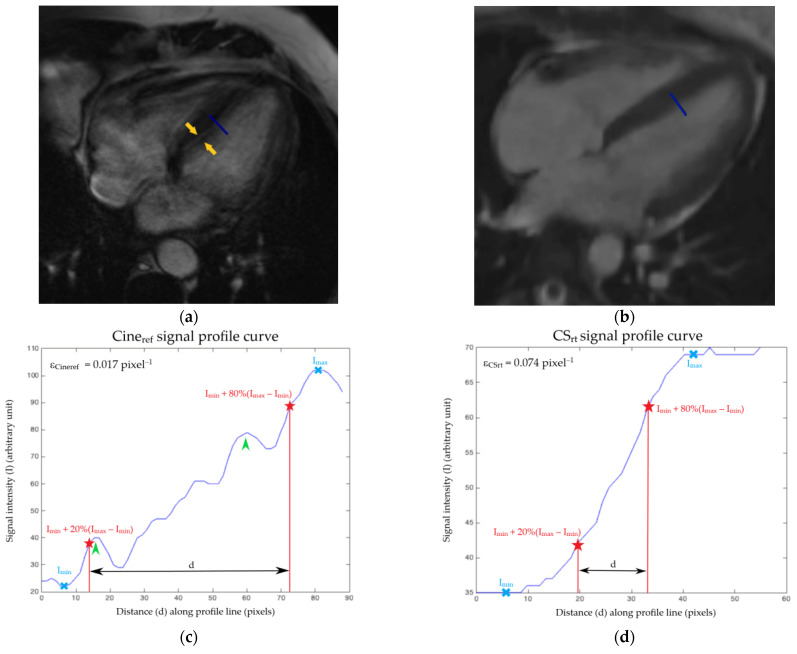
Example of edge sharpness assessment at end-diastole for a 56-year-old male patient suffering from atrial fibrillation. The same four-chamber view at end-diastole is acquired with (**a**) the Cine_ref_ sequence and (**b**) the CS_rt_ sequence. An orthogonal profile line was drawn at mid-cavity across the border between the septal myocardium and the left ventricular blood pool (blue line) on a four-chamber view. It provided intensity profiles (blue curves) along the line for (**c**) Cine_ref_ and (**d**) CS_rt_ cine. The edge sharpness was the inverse of the distance *d* (in pixels) between the positions corresponding to 20% and 80% (red stars) of the difference between the maximum and minimum signal intensities (blue crosses). The edge sharpness was expressed in pixel^−1^. This measurement was performed at end-diastole and end-systole for both sequences. Note that the peaks (arrows heads) added to the Cine_ref_ signal profile curve (**c**) correspond to the doubling of the interventricular septum border (arrows) on the cine view (**a**). The same assessment was performed on both sequences at end-diastole and end-systole for the 71 enrolled patients. Abbreviations: Cine_ref_, reference segmented cine; CS_rt_, real-time compressed sensing cine; ε_Cineref_, edge sharpness measured on Cine_ref_ sequence; ε_CSrt_, edge sharpness measured on CS_rt_ cine; I, signal intensity; I_min_, minimal signal intensity; I_max_, maximal signal intensity; d, distance along the profile line.

Finally, the edge sharpness (ε) of the boundary between myocardium and blood pool, which is the spatial frequency (in pixel^−1^) reflecting the spatial resolution, was measured on paired Cine_ref_ and CS_rt_ four-chamber slices at end-diastole, accordingly to the literature [23,24]. Additional measurement at end-systole was performed. The edge spread function (ESF), which is the response of the imaging system to a high contrast boundary, was measured on MATLAB (version R2015a, The MathWorks, Natick, MA, USA), by drawing a signal profile line perpendicularly across the edge between the interventricular septum and the LV blood pool (Figure 1a,b) [25]. Then, ε was calculated as the reciprocal of the distance separating the points corresponding to 20% and 80% of the difference between local minimum and maximum signal intensities (Figure 1c,d). 

### 2.4. Conditions of Image Analysis 

Images from both sequences were anonymized before transfer to a clinical workstation (Sygno.via VB30A, Siemens Healthcare, Erlangen, Germany). A radiologist with 4 years of experience (PEA) performed the image quality assessment according to the above-cited indicators. Image sets were randomly evaluated in each group. The same observer (PEA) first performed the quality assessment of the reference Cine_ref_ images and at least one month later evaluated the CS_rt_ images. For each patient, arrhythmia was quantified by calculating the CV_RR_. An additional assessment was performed by a radiologist with 8 years of experience (BL) from 30 randomly selected patients to evaluate the interobserver agreement and performed the same assessment regarding subjective quality, EuroCMR score and ARA rates. In the case of mismatch between the two readers, a radiologist with 15 years of experience (FP) performed the quality assessment with the two others to reach consensual scores which were used instead of those set by the first and less experienced observer. Mismatches were defined by discrepancies greater than or equal to 2 points regarding subjective quality score and EuroCMR score, or by a 20% difference in ARA rates. The edge sharpness assessment was automated and was not evaluated for interobserver agreement. Finally, semi-automated segmentation of LV endocardium and epicardium, and manual segmentation RV endocardium were performed on the same workstation with both cine sequences for each patient. LV stroke volume was also measured on PCI sequence.

### 2.5. Statistics Analysis

Categorical data were represented as numbers (percentages), continuous variables as mean ± standard deviation (SD) (95% confidence interval (CI)) in case of normal distribution and median (range: minimum–maximum) in other cases. Sequences were compared using the Wilcoxon signed-rank test regarding the overall subjective quality score and the modified EuroCMR score. Paired Student’s *t*-test was used for ARA rates, edge sharpness comparisons, and ventricular functional parameters comparison. An analysis of variance (ANOVA) was used to compare LV stroke volumes assessed by cine segmentation and PCI flow sequence. Intraclass correlation coefficient and kappa test were applied to assess the interobserver agreement [26]. Values of *p* < 0.05 were considered statistically significant. Statistical analysis was performed using MedCalc software (version 14.8.1.0, MedCalc Software, Ostend, Belgium).

## 3. Results

### 3.1. Population Description

The mean age of the population was 59.5 ± 20.1 (SD) years (95% CI: 54.7–64.2 years) with a male predominance (*n* = 41/71; 57.7%, women: *n* = 30/71; 42.3%). Patients were referred for initial work-up or follow-up of coronary artery disease (*n* = 17; 23.9%), heart rhythm disorder (*n* = 14; 19.7%), dilated cardiomyopathy (*n* = 11; 15.5%), infiltrative cardiomyopathy (*n* = 8; 11.3%), heart valve disease (*n* = 7; 9.9%), myocarditis (*n* = 6; 8.5%), hypertrophic cardiomyopathy (*n* = 5; 7.0%), and heart failure (*n* = 3; 4.2%). The mean HR was 71.8 ± 19.0 beats per minute (bpm) (95% CI: 67.4–76.3 bpm) and 38.0% of the patients (*n* = 27/71) demonstrated a mean HR above 75 bpm, meaning the 49 ms temporal resolution of the CS_rt_ cine provided less than 16 frames of the cardiac cycle per slice. The mean CV_RR_ was 25.0 ± 9.4% (95% CI: 22.8–27.2%). Arrhythmia was caused by atrial fibrillation (*n* = 42/71; 59.2%), ventricular hyperexcitability (*n* = 17/71; 23.9%), and conduction disorders (*n* = 12/71; 16.9%). Demographic data are summarized in Table 3. Biventricular functional assessment of the population is reported in Table 4.

### 3.2. Cine Acquisitions

A total of 599 short-axis cine slices were acquired with each sequence. A median number of 8 (range: 6–14) cine slices was acquired twice for each patient, depending on cardiac morphology. To acquire the same slices, CS_rt_ was significantly faster than Cine_ref_ (Cine_ref_: 556.7 ± 145.4 (SD) s (95% CI: 496.7–616.7 s); CS_rt_: 23.9 ± 7.9 (SD) s (95% CI: 20.6–27.1 s); *p* < 0.0001). 

### 3.3. Objective European CMR Standardized Criteria-Based Quality Score

**Figure 2:**
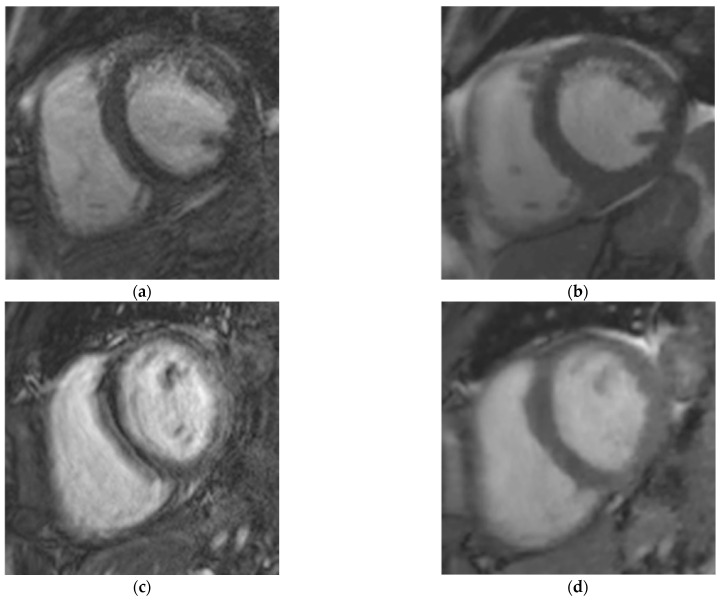

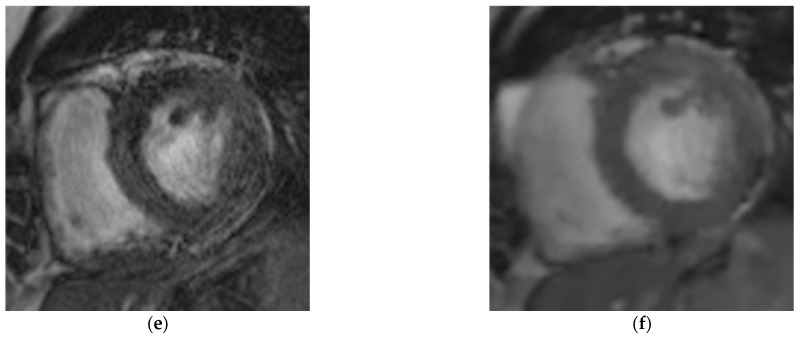
Examples of comparisons between Cine_ref_ and CS_rt_ sequences in three patients suffering from arrhythmia. Mid-cavity short-axis views acquired with (**a**,**c**,**e**) Cine_ref_ and (**b**,**d**,**f**) CS_rt_. The three patients were (**a**,**b**) a 74-year-old man suffering from atrial fibrillation, (**c**,**d**) a 37-year-old woman screened for a genetically proven arrhythmogenic right ventricular cardiomyopathy, (**e**,**f**) a 63-year-old woman scanned for a second-degree atrioventricular block. The image quality assessment demonstrated: (**a**) Likert scale = 1/4, EuroCMR score = 3/10, ε_Cineref_ = 0.051 pixel^−1^; (**b**) Likert scale = 3/4, EuroCMR score = 0/10, ε_CSrt_ = 0.067 pixel^−1^; (**c**) Likert scale = 1/4, EuroCMR score = 3/10, ε_Cineref_ = 0.015 pixel^−1^; (**d**) Likert scale = 3/4, EuroCMR score = 1/10, ε_CSrt_ = 0.050 pixel^−1^; (**e**) Likert scale = 1/4, EuroCMR score = 3/10, ε_Cineref_ = 0.023 pixel^−1^; (**f**) Likert scale = 3/4, EuroCMR score = 0/10, ε_CSrt_ = 0.035 pixel^−1^. Abbreviations: Cine_ref_, reference segmented cine; CS_rt_, real-time compressed sensing cine; ε_Cineref_, edge sharpness measured on Cine_ref_; ε_CSrt_, edge sharpness measured on CS_rt_ cine; EuroCMR, European cardiac magnetic resonance registry.

The EuroCMR score for the CS_rt_ cine (median: 1 (range: 0–5)) was significantly better than for the Cine_ref_ sequence (median: 3 (range: 0–3); *p* < 0.0001) (Table 5) (Figure 2; Video S1 (Supplementary Materials)). Interobserver agreements were 0.94 and 0.89 regarding Cine_ref_ and CS_rt_, respectively. No mismatch was encountered between the readers.

### 3.4. Subjective Overall Quality Score

The subjective quality score was significantly better (*p* < 0.0001) for the CS_rt_ sequence with a median score of 3 (range: 1–3). A 0.85 interobserver agreement was reached. The Cine_ref_ sequence provided a median score of 2 (range: 1–4; *p* < 0.0001) with an intraclass coefficient of 0.82 (Table 6). No mismatch was encountered between the readers. The Cine_ref_ sequence provided 23 non-diagnostic acquisitions compromising functional and morphological assessments versus only 10 stacks with the CS_rt_ cine (Figure 2; Video S1 (Supplementary Materials)).

### 3.5. Arrhythmia-Related Artifacts Rate

ARA using Cine_ref_ sequence were assessed on *n* = 514/599 (85.8%) cine slices from *n* = 70/71 (98.6%) patients, with a 0.90 interobserver agreement. One mismatch was encountered between the readers (PEA: *n* = 8/14, 57.1%; BL: *n* = 12/14, 85.7%; FP: *n* = 12/14, 85.7%). The mean proportion of impaired slices per patient was in 85.9 ± 22.7 (SD) %. No ARA could be depicted using the CS_rt_ sequence.

### 3.6. Edge Sharpness

The CS_rt_ sequence provided a higher edge sharpness coefficient at end-diastole (ε_CSrt_ = 0.051 ± 0.016 pixel^−1^ (95% CI: 0.048–0.055 pixel^−1^)) than the Cine_ref_ (ε_Cineref_ = 0.040 ± 0.018 pixel^−1^ (95% CI: 0.036–0.044 pixel^−1^)) (*p* = 0.0001). A similar finding was observed at end-systole (ε_CSrt_ = 0.054 ± 0.016 pixel^−1^ (95% CI: 0.050–0.057 pixel^−1^); ε_Cineref_ = 0.042 ± 0.022 pixel^−1^ (95% CI: 0.037–0.047 pixel^−1^); *p* = 0.0001). 

## 4. Discussion

This prospective monocentric study based on a 71-patient cohort is, to our knowledge, the widest and most comprehensive study to evaluate the CS_rt_ sequence in patients with irregular HR. Previous studies in non-selected patients confirmed that real-time CS cine imaging is a reliable alternative to segmented multi-breath-hold SSFP for the assessment of both ventricles’ volumes and function in addition to reducing acquisition time [16,17,18,19,20]. Our study demonstrates a dramatic drop in ARA and a significant improvement of subjective and objective image quality with CS_rt_ in patients suffering from heart rhythm disorders. However, no CS_rt_ set was rated as excellent because of the smooth boundaries rendered by the interpolation process which are mandatory for post-processing. Indeed, since the temporal resolution of the CS sequence is fixed, a variable number of frames will be acquired from one cycle to another in the case of arrhythmia. For segmentation to be achieved, post-processing tools require all cine slices to display the same number of frames per cycle. Consequently, a standardization is performed to display 20 frames per cycle on all slices. 

We previously showed on a small sub-group of 25 patients suffering from arrhythmia that CS_rt_ and Cine_ref_ sequences allowed similar image quality [20]. However, the present study does not only suggest equivalent scores but significantly better objective and subjective image quality scores with the CS_rt_ cine. Of note, this sequence still provided non-null EuroCMR scores since the slices were identically located on both sequences; accordingly, most of the wrap-around or metallic artifacts were reproduced on the CS_rt_ acquisition. 

Our results are in line with the previous study by Goebel et al. on 20 patients with atrial fibrillation [21]. This study focused on a subjective semi-quantitative 4-point quality score and the evaluation of the variation of the myocardial signal intensity which is the reciprocal of the signal-to-noise ratio (SNR). However, this last parameter, or its reciprocal, is considered as hardly suitable for non-linear iterative reconstructions. Moreover, CS is built to suppress pieces of the image signal, while SNR is suited for fully sampled data [27]. Besides our study being specifically designed to evaluate the image quality using additional quantitative and objective metrics, we also performed a clinically integrated evaluation in a larger population. 

The higher edge sharpness of CS_rt_ images reflected the faster signal variation along a distance and a better delineation of the image boundaries. We evaluated the edge sharpness both at end-diastole, when the myocardium is supposed to be relatively still, and at end-systole. This metric, regardless of the non-linearity of the reconstruction process, is more suitable than the SNR or its reciprocal to evaluate image quality [27]. The ESF and its inverse value ε measure the imaging system ability to restitute high contrast transition in images. This parameter, the derivative (the line spread function) and its Fourier transform (the so-called task-based modulation transfer function or task transfer function) are currently considered for image quality assessment in the field of non-linear image reconstructions [24,25,28]. 

Regarding volumetric evaluation in patients suffering from arrhythmia, CS_rt_ provided significantly lower LV end-diastolic volume (−3.6 ± 7.2 mL) than measured on Cine_ref_, which was already observed in previous studies [16,20]. As for RV, there was a significant underestimation of all evaluated functional parameters. Nevertheless, these variations compared to the conventional Cine_ref_ should be considered with caution in a population with arrhythmia. Indeed, irregular heartbeats induce variable ventricular preloads and contractions, making the real reference values impossible to determine. Moreover, since CS_rt_ demonstrated a better image quality than Cine_ref_ that was impaired by ARA, one may consider the segmentation to be more reliable on CS_rt_.

Besides the image quality improvement, the single breath-hold CS_rt_ sequence allowed a dramatic reduction in scan time. Not only was acquisition faster, but the ARA reduction avoided repeating the acquisition of non-diagnostic slices [20]. The workflow improvement being a major issue in the field of CMR, this real-time sequence is very promising and may improve cost-effectiveness [29].

### Limitations

Although the overall subjective image quality was improved with CS_rt_ cine, 10 stacks were still considered as non-diagnostic. Indeed, iterative reconstructions occasionally failed or were not completely achieved on this prototype sequence. Nevertheless, such failures are now rare since the release of the final version of the sequence. Moreover, ECG-related issues occurred when R peaks were occasionally missed, which made the system consider two consecutive heartbeats as one single cycle. The corresponding slices then display a double heart cycle which could not be used for post-processing and was ranked as non-diagnostic. Special attention should be paid to skin preparation before ECG electrode placement.

Other fast real-time sequences, such as radial acquisition, have previously been reported [30,31]. Our study does not compare CS_rt_ to other types of real-time sequences. To our knowledge, no such evaluation has been published and further study would be required for comparison.

A methodological limitation of our study is the impossibility to perform blinded evaluation of the sequences since CS_rt_ cine displayed smoother boundaries than Cine_ref_ sequence. Consequently, observers could recognize the type of sequence they were evaluating. However, paired CS_rt_ and Cine_ref_ stacks from the same patients were separated, randomized, and assessed during different sessions.

Regarding the sampling of heart cycles for the assessment of ventricular volumes and mass, the fixed temporal resolution leads to variable sampling rates from shorter cycler to longer ones. As a consequence, in the case of HR faster than 60 bpm, the recommended 20 frames per cycle could not be acquired [9,10]. However, in the field of analog-to-digital signal conversion, a 16-time oversampling is reputed sufficient for the signal restitution to be accurate, corresponding to a 75 bpm HR [32]. High CV_RR_ in HR may be encountered, and some slices may display undersampled heart cycles. In our study, 38% of the patients demonstrated a mean HR above 75 bpm for whom the undersampling of the cardiac cycle should be considered cautiously during interpretation, especially for volume segmentations. Nevertheless, it must be balanced by the reduction of ARA provided by CS_rt_. 

## 5. Conclusions

In addition to reducing acquisition time, CS_rt_ sequence drastically reduces arrhythmia-related artifacts and improves image quality in patients with irregular heart rate. This rapid imaging technique allows practitioners, in daily practice, to improve quality, workflow and accessibility of CMR for patients with challenging cardiac conditions. 

## Figures and Tables

**Table 1:** Imaging parameters of the reference prospectively triggered steady-state free-precession cine imaging and real-time compressed sensing cine imaging.

Parameters	Cine_ref_	CS_rt_
Repetition time—ms	3.16	2.70
Echo time—ms	1.23	1.14
Flip angle—degrees	57	60
Field of view—mm^2^	375 × 280	360 × 270
Matrix—pixels^2^	288 × 216	224 × 168
Spatial resolution—mm^2^	1.3 × 1.3	1.6 × 1.6
Temporal resolution—ms	41.2	49
Slice thickness/gap—mm	8/2	8/2
Bandwidth—Hz/pixel	915	900
ECG mode	Prospective triggering	Adaptative triggering
Number of measured cardiac phases per cycle	20 ^a^	17.0 ± 3.2
Number of reconstructed cardiac frames per cycle—*n*	20 ^a^	20 ^b^
Number of views per frame—*n*	13.0 ± 4.8 ^c^	18 ^a^
Cycles of iterative reconstruction—*n*	NA	40
Acceleration factor	2	11

Data are expressed as mean ± standard deviation in the absence of any indication. ^a^ Constant value. ^b^ Interpolation was performed to provide a constant frame rate of 20 cardiac phases per cycle for post-processing. ^c^ The number of views per frame was set according to the shorter RR interval in order to acquire 20 cardiac phases. Prospective triggering allows data sampling during a fixed acquisition window after each R peak while adaptative triggering allow data sampling until the next R peak occurs. Abbreviations: Cine_ref_, reference segmented cine; CS_rt_, real-time compressed sensing cine; ECG, electrocardiogram; *n*, data represented as numbers; NA, not applicable.

**Table 2:** “LV-Function cine SSFP” section of the standardized objective quality criteria score based on the European CMR registry. Adapted from [22] (p. 3).

Items	0	1	2	3	Maximum Score
**1. LV coverage**	**Full**	**-**	**No apex**	**Base or ≥1 slice missing**	**5**
**2. Wrap around**	**No**	**1 slice**	**2 slices**	**≥3 slices**	**3**
**3. Respiratory ghost**	**No**	**1 slice**	**2 slices**	**≥3 slices**	
**4. Cardiac ghost**	**No**	**1 slice**	**2 slices**	**≥3 slices**	
**5. Blurring/ARA**	**No**	**1 slice**	**2 slices**	**≥3 slices**	
**6. Metallic artifacts**	**No**	**1 slice**	**2 slices**	**≥3 slices**	
**7. Shimming artifacts**	**No**	**1 slice**	**2 slices**	**≥3 slices**	
**8. Signal loss (coil inactive)**	**Activated**	**-**	**Not activated**		**2**
*9. Orientation of stack*	*Correct*	*-*	*Incorrect*	*-*	*2*
*10. Slice thickness*	*≤10 mm*	*11–15 mm*	*-*	*>15 mm*	*3*
*11. Gap*	*≤3 mm*	*3–4 mm*	*-*	*>4 mm*	*3*
*12. Correct LV long axes*	*≥2 mm*	*1*	*-*	*None*	*3*
*Score*					*21*
**Modified score (items 1 to 8)**					**10**

Every acquisition using both sequences marked a null score concerning the four last items. Indeed, acquisitions were repeated every time slice orientation was not appropriated (item 9 = 0); all acquisitions (Cine_ref_ and CS_rt_) were performed using the same slice thickness and gap which were 8 mm (item 10 = 0) and 2 mm (item 11 = 0), respectively, and both horizontal and vertical long-axis slices were systematically acquired (item 12 = 0). Criteria in italics were not applied, and only bold criteria were used for objective quality assessment in our study, providing a maximum score of 10 points. The more artifacts there were, the higher the score was. Abbreviations: LV, left ventricle; SSFP, steady-state free precession; CMR, cardiac magnetic resonance; Cine_ref_, reference segmented cine; CS_rt_, real-time compressed sensing cine; ARA, arrhythmia-related artifacts.

**Table 3:** Study population characteristics.

	Mean ± SD (95% CI)	Minimum Value	Maximum Value
Age—years	59.5 ± 20.1 (54.7–64.2)	18	87
Height—cm	171.6 ± 9.1 (169.4–173.7)	140	188
Weight—kg	79.3 ± 19.5 (74.7–83.9)	26	131
Body mass index—kg/m^2^	26.8 ± 6.1 (25.4–28.3)	13.3	47.0
Maximal heart rate—bpm	85.9 ± 21.6 (80.8–91.0)	50	139
Minimal heart rate—bpm	55.6 ± 18.7 (55.6–64.4)	31	107
Mean heart rate—bpm	71.8 ± 19.0 (67.4–76.3)	42	116
Arrhythmia (CV_RR_)—%	25.0 ± 9.4 (22.8–27.2)	10.2	50.9

Abbreviations: CV_RR_, coefficient of variation of RR interval; bpm, beat per minute; SD, standard deviation; 95% CI, 95% confidence interval.

**Table 4:** Biventricular functional assessment of the study population.

	Cine_ref_	CS_rt_	Difference	PCI	*p*
LVEF—%	47.7 ± 19.0 (39.9–55.6)	47.3 ± 18.9 (39.5–55.1)	−0.4 ± 1.9 (−1.2 to 0.4)	-	0.30 ^a^
LVEDV—mL	193.2 ± 102.0 (151.1–235.3)	189.6 ± 101.9 (147.6–231.6)	−3.6 ± 7.2 (−6.5 to −0.6)	-	0.02 ^a^
LVESV—mL	114.2 ± 99.8 (73.0–155.4)	113.3 ± 98.8 (72.5–154.1)	−0.9 ± 6.8 (−3.7 to 1.9)	-	0.51 ^a^
LVSV—mL	79.0 ± 29.4 (66.9–91.1)	76.3 ± 28.7 (64.5–88.2)	-	76.7 ± 30.1 (64.3–89.1)	0.94 ^b^
LVM—g	145.2 ± 48.0 (125.4–165.1)	148.0 ± 50.1 (127.3–168.6)	2.7 ± 8.8 (−0.9 to 6.3)	-	0.13 ^a^
RVEF—%	50.9 ± 11.9 (46.0–55.8)	51.8 ± 11.9 (46.9–56.7)	0.9 ± 1.8 (0.1 to 1.7)	-	0.02 ^a^
RVEDV—mL	153.7 ± 52.1 (132.2–175.2)	148.4 ± 47.5 (128.8–168.0)	−5.3 ± 7.6 (−8.5 to −2.2)	-	0.02 ^a^
RVESV—mL	77.5 ± 38.0 (61.8–93.1)	73.8 ± 36.1 (58.9–88.7)	−3.7 ± 5.8 (−6.1 to −1.3)	-	0.004 ^a^
RVSV—mL	76.2 ± 27.3 (65.0–87.5)	74.6 ± 24.1 (64.6–84.5)	−1.7 ± 4.5 (−3.5 to 0.2)	Insufficient data	0.08 ^a^

Data are presented as mean ± SD (95% CI). The significance of statistic tests is defined by values of *p* < 0.05. ^a^ Student’s *t*-test; ^b^ Analysis of variance. Abbreviations: Cine_ref_, reference segmented cine; CS_rt_, real-time compressed sensing cine; PCI, phase contrast imaging sequence; SD, standard deviation; 95% CI, 95% confidence interval; LV, left ventricular; RV, right ventricular; EF, ejection fraction; EDV, end-diastolic volume; ESV, end-systolic volume; SV, stroke volume; LVM, left ventricular mass.

**Table 5:** Objective image quality with EuroCMR criteria scores: comparison between Cine_ref_ and CS_rt_ image sets.

Objective European CMR Criteria Scores		CS_rt_					
		0	1–3	4–6	7–10	Total	Median (Range)
Cine_ref_	0	1	1	0	0	2	1 (0–5)
	1–3	25	42	2	0	69	
	4–6	0	0	0	0	0	
	7–10	0	0	0	0	0	
	Total	26	43	2	0	71	
	Median (range)	3 (0–3)					*p* < 0.0001

The significance of Wilcoxon signed-rank test is defined by values of *p* < 0.05. Red values represent patients for whom CS_rt_ score was equivalent to or better than that of Cine_ref_ for *n* = 68/71 patients (95.8%). Abbreviations: CMR, cardiac magnetic resonance; Cine_ref_, reference segmented cine; CS_rt_, real-time compressed sensing cine; EuroCMR, European CMR registry.

**Table 6:** Subjective overall image quality scores: comparison between Cine_ref_ and CS_rt_ image sets.

Subjective Overall Quality Scores		CS_rt_					
		1	2	3	4	Total	Median (Range)
Cine_ref_	1	5	3	15	0	23	3 (1–3)
	2	5	6	21	0	32	
	3	0	2	13	0	15	
	4	0	0	1	0	1	
	Total	10	11	50	0	71	
	Median (range)	2 (1–4)					*p* < 0.0001

The significance of Wilcoxon signed-rank test is defined by values of *p* < 0.05. Red values represent patients for whom CS_rt_ score was equivalent to or better than that of Cine_ref_ for *n* = 64/71 patients (90.1%). Abbreviations: Cine_ref_, reference segmented cine; CS_rt_, real-time compressed sensing cine.

## Data Availability

The data presented in this study are available on reasonable request from the corresponding author, subject to approval by the research ethics committee of Lille University Hospital.

## Supplementary Materials

The following are available online at https://www.mdpi.com/article/10.3390/jcm10153274/s1, Figure S1: Study design. Video S1: Viability assessment in a 77-year-old man with premature ventricular contractions caused by ischemic scars.
