# Supplementary material for: Compressed Sensing Real-Time Cine Reduces CMR Arrhythmia-Related Artifacts
Source: J Clin Med. 2021 Jul 24;10(15):3274. doi: 10.3390/jcm10153274 (PMC8348071; doi:10.3390/jcm10153274)
Supplement: Supplementary file 1 [file jcm-10-03274-s001.zip › Figure S1 - Study design.pdf]

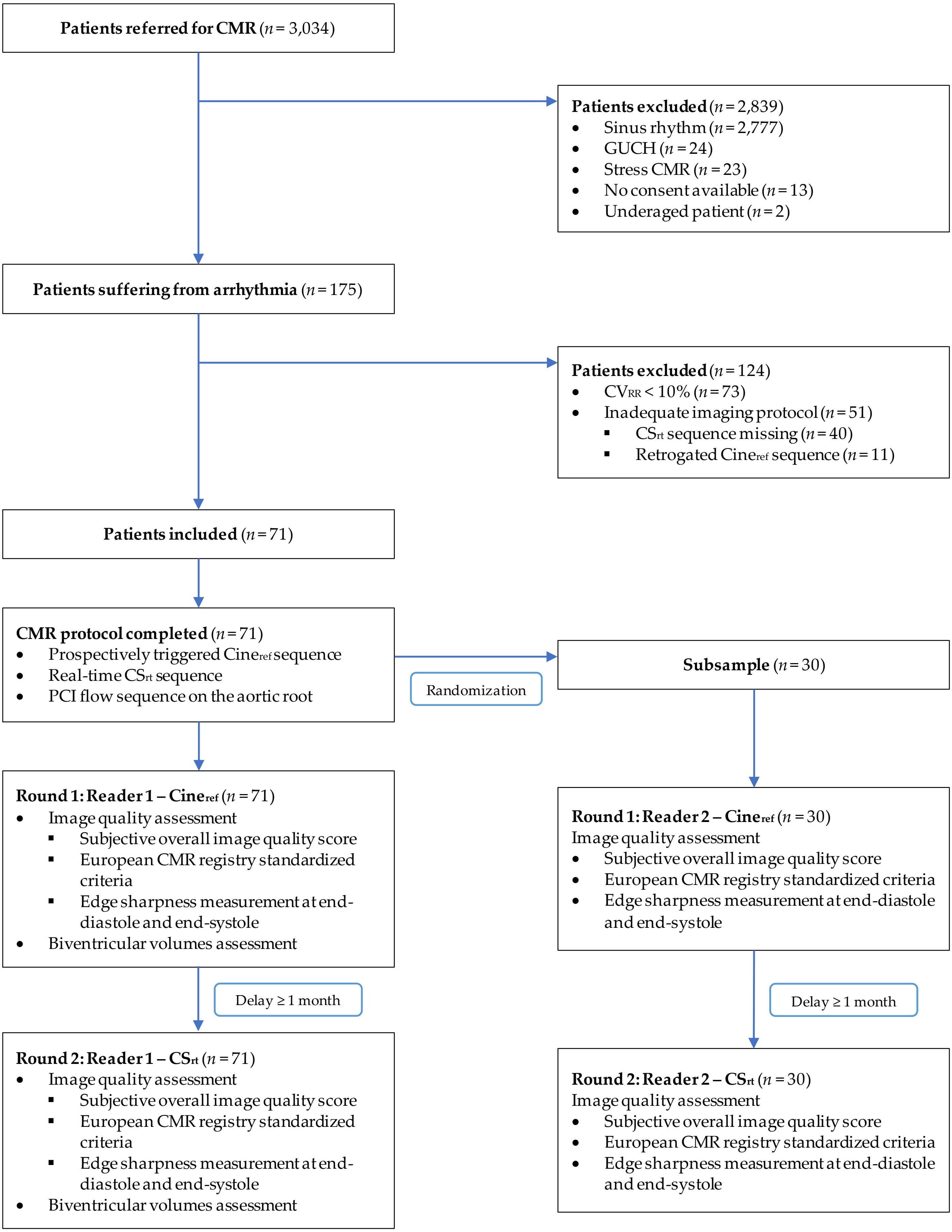

Figure S1: Study design. Student's t test and analysis of variance were used for volumes assessments comparisons. Wilcoxon signed-rank test was used for subjective and EuroCMR quality scores. Student's t test was used for edge sharpness comparison. Intraclass coefficient correlation assessed the interobserver agreement.

Abbreviations: GUCH, grown-up congenital heart disease; CMR, cardiac magnetic resonance; CV_{RR}, coefficient of variation of RR intervals; CS_{rt}, real-time compressed sensing cine; $Cine_{ref}$, reference prospectively triggered segmented cine; PCI, phase contrast imaging; EuroCMR, European CMR registry.
